# A Direct Immunoassay Based on Surface-Enhanced Spectroscopy Using AuNP/PS-b-P2VP Nanocomposites

**DOI:** 10.3390/s23104810

**Published:** 2023-05-16

**Authors:** Moyra F. Vieira, Ana Lívia de Carvalho Bovolato, Bruno G. da Fonseca, Celly M. S. Izumi, Alexandre G. Brolo

**Affiliations:** 1 Department of Chemistry and Center for Advanced Materials and Related Technologies, University of Victoria, P.O. Box 3065, Victoria, BC V8W 3V6, Canada; 2 Departamento de Química, Instituto de Ciências Exatas, Universidade Federal de Juiz de Fora, Campus Universitário s/n, CEP, Juiz de Fora 36036-900, Brazil

**Keywords:** polystyrene-b-poly(2-vinylpyridine), gold nanoparticles, LSPR sensor, SERS sensing, direct immunoassay

## Abstract

A biosensor was developed for directly detecting human immunoglobulin G (IgG) and adenosine triphosphate (ATP) based on stable and reproducible gold nanoparticles/polystyrene-b-poly(2-vinylpyridine) (AuNP/PS-b-P2VP) nanocomposites. The substrates were functionalized with carboxylic acid groups for the covalent binding of anti-IgG and anti-ATP and the detection of IgG and ATP (1 to 150 μg/mL). SEM images of the nanocomposite show 17 ± 2 nm AuNP clusters adsorbed over a continuous porous PS-b-P2VP thin film. UV–VIS and SERS were used to characterize each step of the substrate functionalization and the specific interaction between anti-IgG and the targeted IgG analyte. The UV–VIS results show a redshift of the LSPR band as the AuNP surface was functionalized and SERS measurements showed consistent changes in the spectral features. Principal component analysis (PCA) was used to discriminate between samples before and after the affinity tests. Moreover, the designed biosensor proved to be sensitive to different concentrations of IgG with a limit-of-detection (LOD) down to 1 μg/mL. Moreover, the selectivity to IgG was confirmed using standard solutions of IgM as a control. Finally, ATP direct immunoassay (LOD = 1 μg/mL) has demonstrated that this nanocomposite platform can be used to detect different types of biomolecules after proper functionalization.

## 1. Introduction

Biosensors based on localized surface plasmon resonance (LSPR) are widely studied due to their ability to detect trace levels of analytes in a simple, sensitive, and rapid manner [1]. Noble metal nanoparticles, such as gold nanoparticles (AuNPs), exhibit a distinctive extinction band in their optical spectrum due to the LSPR phenomenon. LSPR is generated by the coherent collective oscillation of the conduction electrons of these particles when excited by electromagnetic radiation [2]. These oscillations (LSPR) lead to large local electrical field enhancements that are enhanced in small aggregates of metallic nanoparticles due to strong short-range electromagnetic interactions. The localized regions of high electric fields are known as hotspots [3]. Therefore, molecules adsorbed onto the surfaces of these plasmonic structures experience the amplification of their Raman scattering signal. Moreover, the LSPR peak extinction wavelength, λ_max_, and the SERS signal strongly depend on the characteristics of the AuNPs, including their dielectric properties, size, shape, and interparticle distance [4]. LSPR is also sensitive to the local environment, including the solvent and any layer of adsorbates in contact with the metal surface [5]. Therefore, a shift in the LSPR peak can be induced in combination with changes in the SERS spectral features in response to the binding of specific targets to the NPs. Hence, by appropriately functionalizing the AuNPs surfaces, it is possible to design LSPR and SERS sensors capable of detecting various biological and pathogenic species [6,7,8].

AuNPs in colloidal suspensions have been extensively used as LSPR and SERS biosensors because many synthetic routes yield materials with a controlled shape, size distribution, and chemical composition. In addition, the large surface:volume ratio of metallic nanoparticles provides strong binding sites for anchoring molecules [9]. However, the applications of colloidal sensors are limited by their poor stability over time and reproducibility [10].

Consequently, many studies have focused on synthesizing templates for AuNPs immobilization on solid substrates [1,11,12]. In contrast to the direct use of colloidal suspensions, the immobilized NPs would be resistant to aggregation, leading to an overall longer time stability. However, to date, the construction of reliable and reproducible SERS substrates is still challenging because of the difficulty in immobilizing large areas of plasmonic-active nanoparticles [13]. The use of a block copolymers (BCPs) template is one of the most efficient approaches for the stabilization, design, and fabrication of large-area arrays of NPs, due to their ability to self-assemble in different morphologies at the nanoscale, such as lamellar, gyroid, spherical, and cylindrical [14,15,16,17].

Although previous studies have applied poly(styrene-block-2-vinylpyridine) (PS-b-P2VP) as a template for the adsorption of pre-synthetized nanoparticles [18,19], their application as biosensors is yet to be demonstrated. In a previous study, we developed a straightforward method to synthesize stable and reproducible large-area substrates with tunable surface-enhanced Raman scattering (SERS) enhancement from self-assembled citrate-stabilized AuNPs in reconstructed polystyrene-b-poly(2-vinylpyridine) (PS-b-P2VP) films (AuNP/PS-b-P2VP) [20]. We have demonstrated that the relative volume fraction of PS/P2VP and the annealing treatments strongly influence the morphologies of the films, allowing the modulation of density, distribution, and optical properties of the nanocomposites. That was one of the first studies to quantify these parameters systematically, which is essential to developing a sensitive SERS-based biosensor [20].

This work presents the development of a direct label-free SERS-based sensor for the detection of a model immunoglobulin G (IgG) based on stable and reproducible SERS substrate. IgG is the most common antibody in humans and its concentration in healthy adults varies from 7 to 18 mg/mL depending on the response against different pathogens, such as viruses and bacteria [21]. The fabrication and detection of various concentrations of IgG (1 to 150 μg/mL) were achieved by step-by-step functionalization of the AuNPs/PS_1250_-b-P2VP_1285_ with anti-IgG and applying SERS spectroscopy and principal component analysis (PCA) analysis. Additionally, to verify the selectivity of the biosensor, a standard IgM solution was used as a negative control. In order to demonstrate that the approach reported here can be adapted to detect different types of analytes, the AuNPs/PS_1250_-b-P2VP_1285_ system was also modified with anti-ATP (anti-adenosine triphosphate), and an assay for the direct detection of ATP, an important biomarker for Alzheimer [22], was developed.

## 2. Materials and Methods

### 2.1. Chemicals

PS_1250_-b-P2VP_1285_ (Mn = 265,000 g/mol) was purchased from Polymer Source, Inc. (Dorval, QC, Canada). Tetrachloroauric acid (HAuCl_4_), phosphate buffered solution (PBS in 0.1 mol L^−1^ at pH 7.4), bovine serum albumin (BSA), 11-mercapto undecanoic (11-MUA), 3-mercapto propionic acid (MPA), N-(3-dimethylaminopropyl)-N′-ethyl carboiimide hydrochloride (EDC), N-hydroxysuccinimide (NHS), immunoglobulin G (IgG) from human serum, and anti-human immunoglobulin G (anti-IgG) were purchased from Sigma Aldrich (St. Louis, MO, USA). Adenosine triphosphate (ATP) synthase subunit beta and mitochondrial and anti-adenosine triphosphate antibody (anti-ATP) were purchased from MyBioSource. All solvents were provided by Sigma Aldrich, USA. All reagents were used without any further purification.

### 2.2. Preparation of the Glass and Gold Slides

The 100 nm gold-coated glass slides (2 cm × 2 cm) were cleaned by the adaptation of a procedure previously reported in the literature [23]. The substrates were cleaned in an ultrasonic bath with ethanol, isopropyl alcohol, and acetone for 10 min each. Afterward, the substrates were immersed in piranha solution (H_2_SO_4_/H_2_O_2_ = 7:3) for 30 min, rinsed thoroughly with deionized water, and dried under ambient conditions.

### 2.3. Preparation of AuNP/PS-b-P2VP Nanocomposites

The AuNP/PS-b-P2VP nanocomposites were prepared as previously described. Briefly, PS-b-P2VP in toluene (0.5 wt%) was spin-coated onto glass and gold substrates at 3000 rpm for 60 s. The films were treated with deionized water for 10 min and then immersed in gold nanoparticle solution for 6 h. The colloidal suspension was synthesized using Turkevich’s method and adapted by Frens and co-workers [24,25].

### 2.4. Thin Film Functionalization

The fabricated substrates were functionalized with carboxylic acid groups for the covalent binding with the antibodies by adapting a procedure described in the literature [26]. The substrates were first treated with 20 mM of 11-MUA/MPA (1:9) for 18 h at room temperature, followed by extensive washing with ethanol to remove any excess of 11-MUA/MPA. Additionally, to activate the carboxylic acid groups inserted in the previous step, the substrates were treated with a 1:1 solution of EDC (0.15 M) and NHS (0.03 M) in PBS for one hour at room temperature. Finally, the substrates were extensively washed with PBS to remove the excess EDC/NHS.

### 2.5. Antibody/Antigen Biding

The substrates were treated with a solution of standard anti-IgG suspended in PBS (150 µg/mL) for three hours at 4 °C. After, the substrates were washed with PBS to remove the excess of anti-IgG. To block any unbound sites in the AuNPs surfaces, the substrates were treated with BSA (100 µg/mL) overnight at 4 °C. Then, the substrates were incubated in solutions of IgG in PBS (concentrations from 1 to 150 μg/mL) for two hours at room temperature. Finally, the substrates were washed with PBS to remove the excess of antigen.

After each step of the substrate preparation, absorbance spectra and SERS mapping were acquired to confirm the functionalization and the detection of the antigen.

### 2.6. Control Experiment

For the control experiment, the substrates functionalized with anti-IgG were incubated in a solution of IgM in PBS (100 µg/mL) for one hour at room temperature. The substrates were washed in PBS and dried under N_2_ flow.

### 2.7. ATP Direct Immunoassay

For the ATP direct immunoassay, the nanocomposites were functionalized following the steps described in Section 2.2. Afterward, the substrates were incubated in a solution of anti-ATP in PBS (100 μg/mL) for one hour at room temperature and treated with solutions of ATP (concentration range: 1 to 100 μg/mL).

### 2.8. Characterization

UV–VIS absorption spectra were obtained using a Perkin Elmer Lambda 1050 spectrophotometer equipped with a 1.0 cm silica cell. The films were deposited on glass slides and they were fixed onto the sample holder. A clean glass slide of the same dimensions was used as a reference. The spectra were recorded in a range of 340–1100 nm.

Scanning electron microscopy (SEM) images were recorded in a Hitachi S-4800 SEM microscope with an acceleration voltage of up to 30 kV. TEM images were recorded on an FEI Tecnai Spirit transmission electron microscope with an acceleration voltage of 120 kV (INMETRO, Duque de Caxias, Brazil). The average diameter of the synthesized gold nanospheres was calculated by measuring the size of 250 nanoparticles in the TEM images (Supplementary Information—Figure S1) using Image J software.

Raman analysis and SERS mappings were carried out using a Renishaw inVia Raman microscope system with an excitation laser at 785 nm, 20× objective lens, and an exposure time of 3 s. The SERS mappings were performed in 10 μm × 10 μm area. For the SERS mapping of the AuNPs/PS_1250_-b-P2VP_1285_ substrate functionalization experiment, 100 spectra were collected; 2500 spectra were registered for the detection of different concentrations of IgG and 1350 spectra were collected for ATP direct immunoassay. All spectra were smoothed, the baseline corrected and normalized, and then analyzed with PCA using MATLAB software (The Mathworks, Natick, MA, USA). PCA is an unsupervised model that simplifies the visualization of high dimensional data into smaller datasets known as principal components (PCs) while maintaining most of the original spectral features and reflecting the difference between the samples [26].

## 3. Results and Discussions

### 3.1. PS-b-P2VP Thin Film Characterization

**Figure 1:**
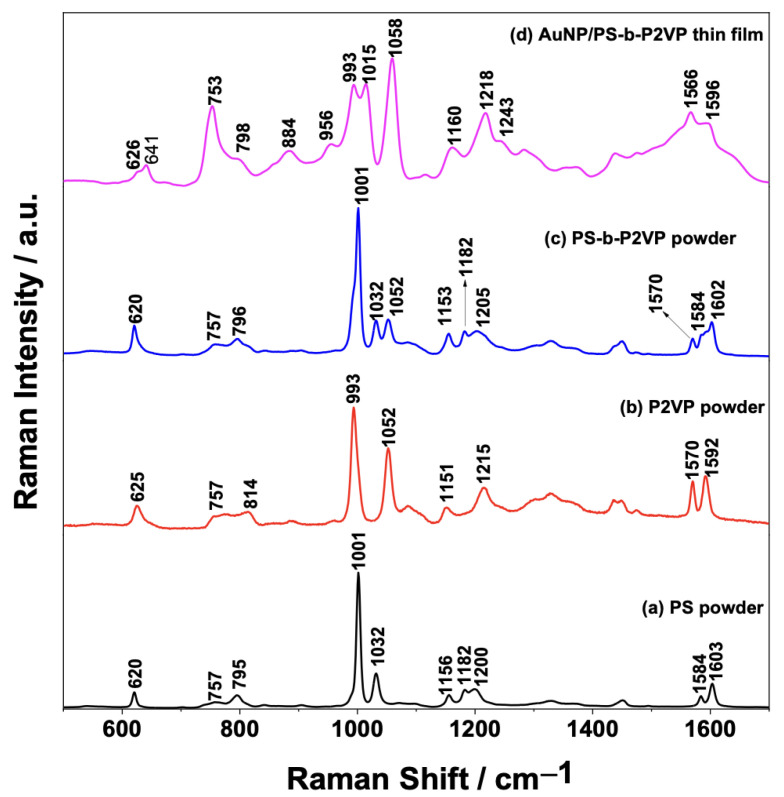
Comparison between the Raman spectra of the solid (a) PS, (b) P2VP, and (c) PS-b-P2VP powders and (d) AuNP/PS_1250_-*b*-P2VP_1285_ thin film (SERS spectrum in this case).

Figure 1 shows the mean Raman spectra of the solid PS (Figure 1a), P2VP (Figure 1b), and PS-b-P2VP (Figure 1c) powders as well as the SERS spectrum of the AuNP/PS-b-P2VP nanocomposite (Figure 1d). Particular emphasis was placed on the strong band observed in 1001 cm^−1^ in the normal Raman spectrum of PS (Figure 1a), which is assigned to ν(1), a totally symmetric ring breathing mode, and the medium-intensity band in 1032 cm^−1^ assigned to ν(18a) related to in-plane bending. As the homopolymers have some structural similarities, the Raman spectra share some common modes at similar wavenumbers. However, differences can also be observed, for example, the band in 1052 cm^−1^ in the P2VP Raman spectrum (Figure 1b) is assigned to a CH in-plane breathing mode ν(18b) [27,28]. The intensity of this ν(18b)-band is higher in the solid P2VP than in the PS-b-P2VP (Figure 1c). The result in Figure 1 clearly confirms the composition of the copolymer since the Raman spectrum of PS-b-P2VP is a combination of the spectra of the blocks (PS and P2VP).

The thickness of the PS-b-P2VP thin films were controlled by spin coating [29]. The normal Raman spectrum of the PS-b-P2VP thin film is shown in the Supplementary Information (Figure S2). Unfortunately, it was not possible to observe any of the PS-b-P2VP characteristic Raman bands due to the small thickness of the thin films and the low scattering cross-section of the copolymer. Figure 1d shows the SERS spectrum for the AuNP/PS-b-P2VP thin films. It is important to notice that the band assigned to the ν(18a) mode on the ordinary Raman spectra of the nanocomposites (1032 cm^−1^ band in Figure 1c) downshifted to 1015 cm^−1^ in Figure 1d. Moreover, the bands around 753, 1015, and 1058 cm^−1^, due to the ν(13), ν(18a), and ν(18b) modes, are relatively more intensified than the ring breathing (ν(1) mode) at ~993 cm^−1^. Figure 1d shows that the adsorption of AuNPs over the thin PS-b-P2VP film leads to a strong intensification of the Raman signal (compared to the normal Raman of the film in Figure S2) and that the ring structure is significantly altered by the interaction between the pyridine ring and the AuNPs [27,28,30].

**Figure 2:**
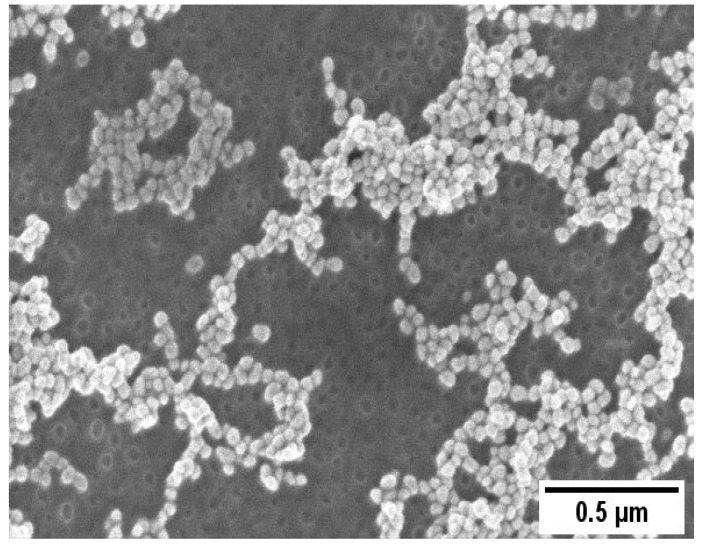
SEM images of the AuNP/PS_1250_-b-P2VP_1285_.

A representative SEM image of the AuNPs/PS_1250_-b-P2VP_1285_ thin film is shown in Figure 2. AuNPs with an average size of (17 ± 2 nm) adsorbed over a continuous porous PS_1250_-b-P2VP_1285_ thin film are evident in Figure 2. Amphiphilic BCPs dissolved in nonpolar solvents (e.g., toluene) and above the critical micelle concentration self-assemble in reverse micelles consisting of a core composed by the hydrophilic block and a soluble corona formed by the hydrophobic block. These micelles can be transferred to a substrate via spin coating, creating a thin film of packed micelles [31,32,33]. A water-induced reorganization of the polymer morphology occurs upon immersion of the PS_1250_-b-P2VP_1285_ micellar film in water. The overall process has two steps: diffusion of water through the PS coronae and swelling of the P2VP. The rupture of the micelles occurs when certain critical moment conditions are achieved, exposing the P2VP block. Since the P2VP has a stronger affinity to the solvent, the P2VP chains move outward from the micelle center and merge, shielding the PS block underneath from contacting the water to reduce the interfacial energy and, consequently, creating holes [16].

### 3.2. Preparation of the Biosensor

**Figure 3:**
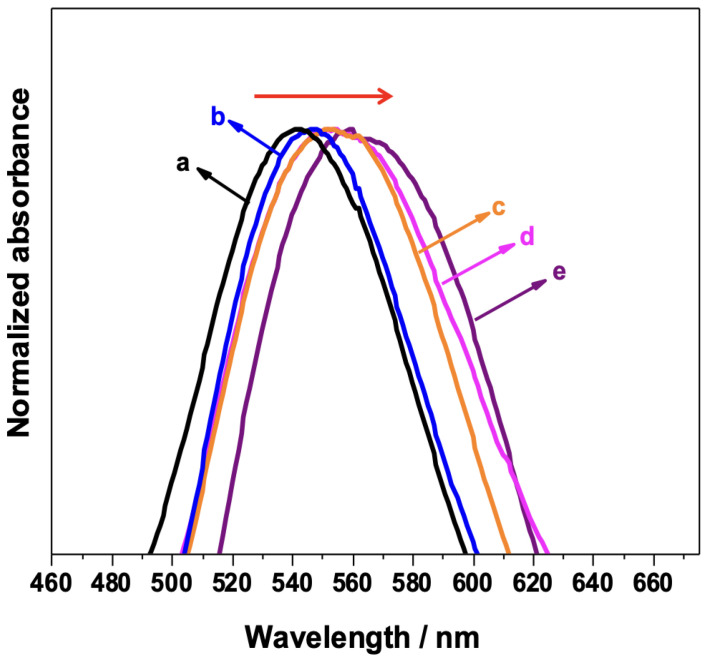
UV–VIS spectra of each step of the AuNPs/PS_1250_-b-P2VP_1285_ substrate functionalization: (a) AuNP/PS-b-P2VP; (b) AuNP/PS-b-P2VP functionalized with MUA/MPA; (c) AuNP/PS-b-P2VP/MUA/MPA further functionalized with EDC/NHS; (d) AuNP/PS-b-P2VP/MUA/MPA/EDC/NHS functionalized with anti-IgG; and (e) full sensor (indicated in (d)) exposed to 150 μg/mL IgG.

The fabrication of the biosensor for detecting the target antigen was achieved by step-by-step functionalization of the AuNPs/PS_1250_-b-P2VP_1285_. It is well established that the position of the LSPR maximum (λ_max_) is affected by changes in the interfacial refractive index induced by ligand binding events [8,34,35]. Therefore, each functionalization step induces different dielectric environments at the Au/ligand interface, leading to changes in the spectral features of the AuNPs. The normalized UV–VIS spectrum of each step of the functionalization is shown in Figure 3. As the AuNP surface is functionalized, a redshift of the λ_max_ is observed, confirming the functionalization. A redshift is also induced by antigen binding when the platform (AuNPs/PS_1250_-b-P2VP_1285_) functionalized with anti-IgG is used as a biosensor.

**Figure 4:**
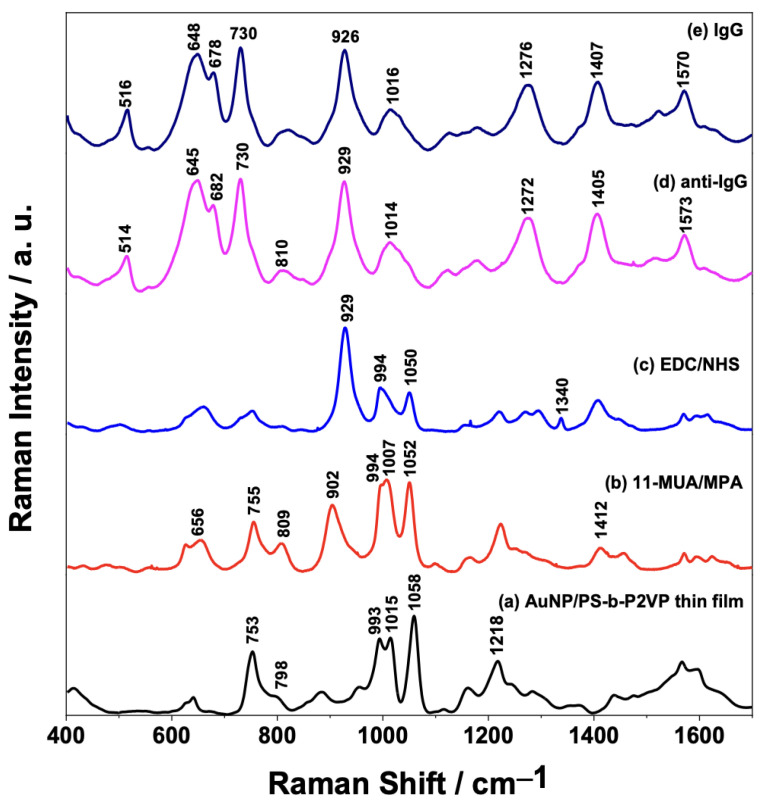
Average SERS spectra (*n* = 100 spectra) of each step of the AuNPs/PS_1250_-b-P2VP_1285_ substrate functionalization and detection of the interaction between anti-IgG/IgG: (a) AuNP/PS-b-P2VP; (b) AuNP/PS-b-P2VP functionalized with MUA/MPA; (c) AuNP/PS-b-P2VP/MUA/MPA further functionalized with EDC/NHS; (d) AuNP/PS-b-P2VP/MUA/MPA/EDC/NHS functionalized with anti-IgG; and (e) full sensor (indicated in (d)) exposed to 150 μg/mL IgG.

Figure 4 shows the mean SERS spectra of the AuNPs/PS_1250_-b-P2VP_1285_ film after each functionalization step (Figure 4a–d). The SERS spectrum of the fully assembled sensing surface (containing anti-IgG) after interaction with the IgG analyte (150 μg/mL) is also present in Figure 4e. The presence of the characteristic peaks at 656 cm^−1^ due to ν(CS) and 902 cm^−1^ assigned to ν(C-COOH) mode in Figure 4b suggests that the AuNPs/PS_1250_-b-P2VP_1285_ substrate was successfully modified with 11-MUA/MPA [36].

The carboxylic acid moiety of the 11-MUA/MPA was activated by treating the substrate with EDC/NHS to enable antibody immobilization onto the AuNPs. The conversion of the -COOH group to a succinimidyl ester allows the attachment of the antibody by a nucleophilic attack from the amine groups of the lysine residues of the antibody, as demonstrated by Hibbert and coworkers [37,38]. The mean SERS spectrum presented in Figure 4c shows that the band at 902 cm^−1^ in 11-MUA/MPA (Figure 4b), assigned to the ν(C-COOH) mode, is shifted to 929 cm^−1^ after the activation step. Moreover, the presence of this band in the SERS spectra of anti-IgG (Figure 4d) and IgG (Figure 4e) could be related to the amide group formed after the covalent attachment of the antibody to the surface of the AuNPs. After the antibody binding step to AuNPs, BSA was used as a blocking agent to block the remaining free sites on the Au surface and prevent further non-specific binding [39,40,41]. BSA binds spontaneously to the surface of citrate-stabilized AuNPs through electrostatic interactions between the negative charge from the citrate and the positive charge due to the lysine groups of BSA [42]. Typical vibrational frequencies of proteins are mostly correlated with the functional groups in amino acids, backbone structures, and side chains environment, in addition to the specific secondary structural element [43]. Thus, it is expected that the specific interaction between the IgG and the immobilized anti-IgG antibody would cause spectral differences, such as changes in relative intensity ratios and frequency shifts. However, by only comparing the averaged SERS spectra of the sensor surface before and after the assay (IgG binding—Figure 4d,e) is not possible to observe significant spectral changes, so a more sophisticated statistical analysis is required.

**Figure 5:**
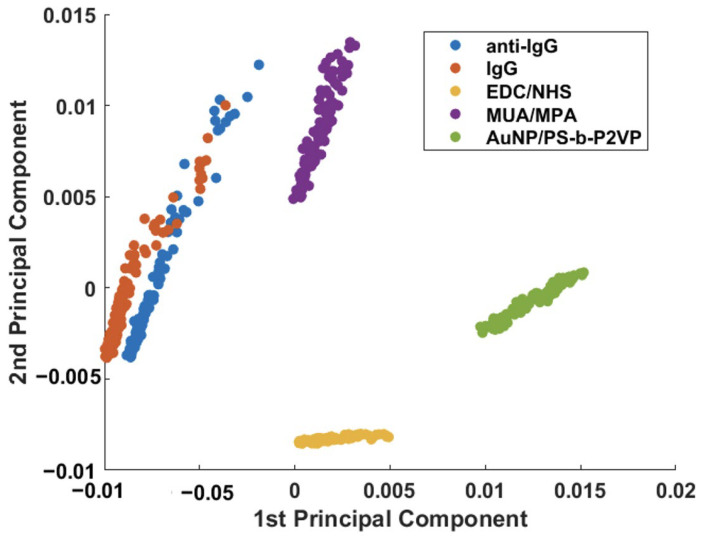
Binary scatterplot of the PC1 × PC2 scores of functionalization steps and IgG detection (150 μg/mL).

PCA was applied to the dataset in an attempt to unveil the small spectral differences in the immunoassay (anti-IgG–IgG interaction). Figure 5 shows the score plot for PC1 and PC2 obtained by combining all SERS spectra obtained after each modification step (Figure 3 and Figure 4). From the plot, it is possible to observe a clear discrimination between each step of the AuNPs/PS-b-P2VP substrate functionalization. However, some overlap between PC1 and PC2 scores of the substrate modified with anti-IgG before and after the interaction with IgG (immunoassay) is still observed.

**Figure 6:**
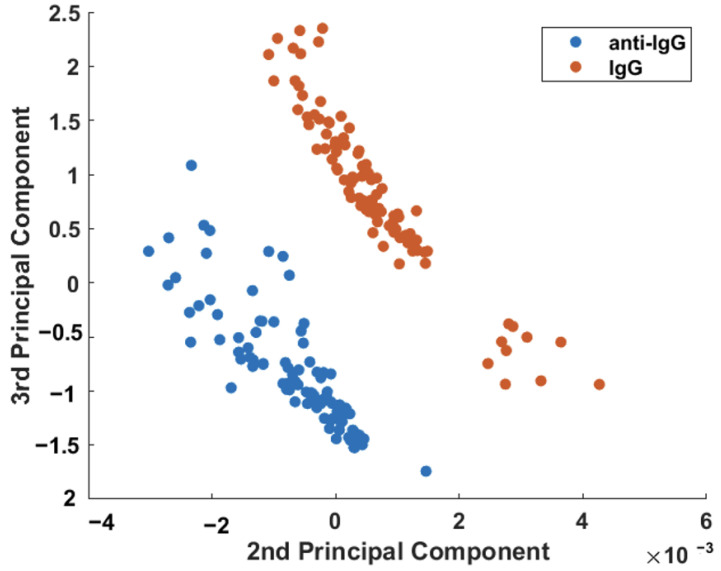
Binary scatterplot of the PC2 × PC3 scores for better discrimination between the sensor before (anti-IgG) and after exposure to 150 μg/mL of the IgG analyte.

Figure 6 shows that a binary scatterplot of the PC2 × PC3 scores, which account for 8.68% of the total variation in the dataset (Supplementary Information—Figure S3), provides much better discrimination between the sensor before (anti-IgG) and after exposure to the IgG analyte. It is possible to clearly observe the separation between the two samples from the plot in Figure 6.

### 3.3. Detection of Different Concentrations of IgG

**Figure 7:**
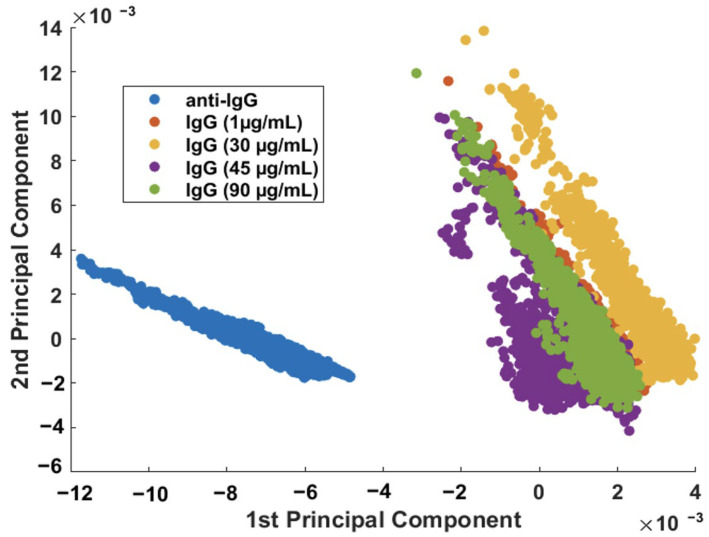
Binary scatterplot of the PC1 × PC2 scores of the SERS spectra (*n* = 2500 spectra) of anti-IgG and IgG samples (concentration range: 1 to 90 μg/mL).

Figure 6 showed that it is possible, using PCA, to discriminate the SERS characteristics of the sensor before and after the adsorption (capture) of the analyte (IgG). The next step was to verify the response of the biosensor to different concentrations of the IgG analyte. The sensor surface (nanocomposite functionalized with anti-IgG, as discussed in Figure 3 and Figure 4) was incubated in various IgG solutions with concentrations ranging from 1 to 90 μg/mL, followed by the SERS measurement (Supplementary Information—Figure S4). From the scatterplot of the PC1 × PC2 scores shown in Figure 7, it is possible to conclude that the IgG analyte can be easily discriminated from the sensor at every concentration investigated here.

It is well stablished that conventional methods for IgG identification based on antigen–antibody coupling, such as enzyme-linked immunosorbent assays (ELISA), have high specificity and selectivity with LOD down to nanomolar concentration levels. However, these methods involve laborious procedures, have limited multiplexing options, require expensive laboratory equipment, and need a relatively high sample volume [44,45]. Moreover, the results described here confirm that the AuNPs/PS_1250_-b-P2VP_1285_ nanocomposite modified with anti-IgG can be used for direct label-free immunoassays by Raman spectroscopy with good sensitivity down to 1 μg/mL. Moreover, we believe that the LOD could be even lowered with further optimization of the nanocomposite’s functionalization process.

Although the SERS procedure described can detect IgG down to 1.0 μg/mL (unoptimized), the results in Figure 7 suggest that the different concentrations of IgG cannot be separated; therefore, the protein cannot be quantified using this procedure. While quantification was not possible, the developed biosensor is still useful as a screening tool.

### 3.4. Selectivity Assay

**Figure 8:**
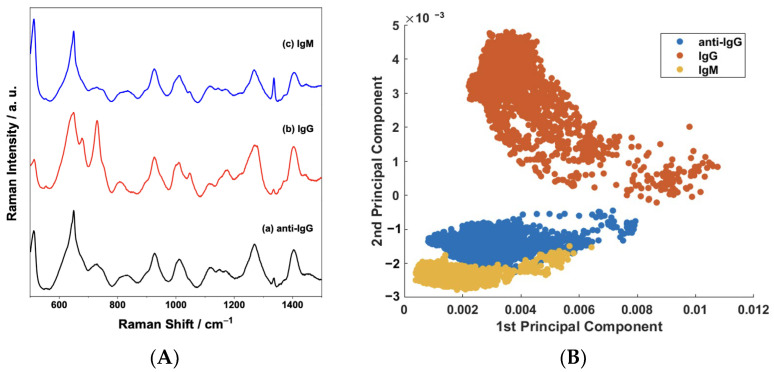
(**A**) Comparison between the mean SERS spectra of the sensor before (anti-IgG) and after exposure to 100 μg/mL of the IgG and IgM analytes; (**B**) binary scatterplot of the PC1 × PC2 scores of the SERS spectra of anti-IgG and IgG and IgM samples.

The selectivity of the biosensor to IgG was tested by devising a negative control experiment using standard IgM solution due to its abundant presence in the human blood plasma (40 to 230 mg/dL) [43]. Figure 8A presents the average SERS spectra of the sensor surface before and after being exposed to either IgG or IgM. The spectral features in Figure 8A—(a), such as changes in relative intensity ratios and frequency shifts, do not significantly change when the sensor is challenged by IgM (Figure 8A—(c)). On the other hand, obvious changes are present after the selective binding interaction with IgG (Figure 8A—(b)). The good discrimination and selectivity are confirmed in the scatterplot of the PC1 × PC2 scores shown in Figure 8B. PCA enables the investigation of spectral variations, allowing the differentiation between samples [46]. Hence, the overlap between IgM and anti-IgG samples in the plot confirms a small variability in spectral features, implying that no important interaction occurs between the anti-IgG and IgM.

### 3.5. ATP Direct Immunoassay

**Figure 9:**
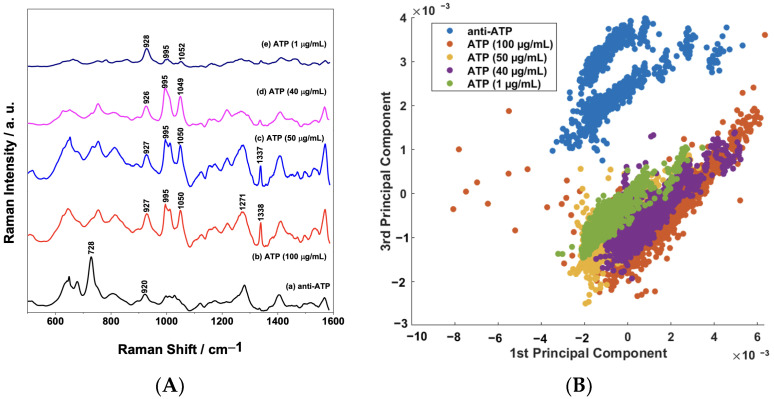
(**A**) Comparison between the mean SERS spectra (*n* = 1350 spectra) of anti-ATP and ATP samples (concentration range: 1 to 100 μg/mL); (**B**) binary scatterplot of the PC1 × PC3 scores of the SERS spectra of anti-ATP and ATP samples.

The assay platform based on AuNPs/PS_1250_-b-P2VP_1285_ can be extended to detect different types of biomolecules by simply adjusting the functionalization strategy. In order to demonstrate this advantage, an assay for direct label-free detection of ATP at different concentrations (1 to 100 μg/mL) was executed. Figure 9A shows a comparison of the mean SERS spectra of the sensor surface before and after exposure to ATP samples of different concentrations. It is possible to observe several variations in spectral features due to the binding of the analyte (ATP). It is well-established that the spectral features of the SERS spectrum of the ATP molecule can be assigned to the adenine, ribose, and phosphate groups [47]. Therefore, in the SERS spectra of the ATP shown in Figure 9A, the bands around 928 and 995 cm^−1^ could be assigned to the stretching vibration of a phosphate group and the band at 1049 cm^−1^ is assigned to the symmetric stretch of O=P=O. Moreover, the bands in the region between 1200 and 1600 cm^−1^ are mostly due to vibrations of the adenine moiety [48,49].

The PCA of the biosensor SERS response before and after binding is presented in Figure 9B. The binary scatterplot of the PC1 × PC3 scores, which account for 58.66% of the total variation in the dataset (Supplementary Information—Figure S5) in Figure 9B, shows a good separation between the samples, as observed previously in Figure 7. These results confirm that the functionalization of the AuNPs/PS_1250_-b-P2VP_1285_ nanocomposite can be tailored to detect different types of biomolecules with good sensitivity.

## 4. Conclusions

In the present work, we demonstrated a straightforward method to construct a sensitive and selective biosensor based on the self-assembled gold nanoparticles in PS-b-P2VP thin film. The analysis using UV–VIS spectroscopy confirmed the step-by-step functionalization through the consistent redshift of the maximum of the LSPR band. The changes in the SERS spectral features after each functionalization step corroborate the UV–VIS results. The designed biosensor proved to be sensitive to different concentrations of IgG (1 to 150 μg/mL), as shown in the SERS and PCA results. The selectivity of the assay was confirmed by a negative control using IgM. Finally, an ATP label-free immunoassay was developed to demonstrate that the AuNPs/PS_1250_-b-P2VP_1285_ nanocomposite can be adapted to detect different types of biomolecules by changes in the functionalization process with LOD down to 1 μg/mL. Thus, the designed label-free SERS-based sensor is expected to be an attractive clinical tool to be implemented in the detection of biological species.

## Data Availability

The data presented in this study are available in the article and Supplementary Materials.

## Supplementary Materials

The following supporting information can be downloaded at: https://www.mdpi.com/article/10.3390/s23104810/s1, Figure S1. A TEM image of the synthesized citrate stabilized gold NPs. Figure S2. Raman spectra of the PS_1250_-*b*-P2VP_1285_ thin film. Figure S3. PCA of functionalization steps and IgG detection: (a) Explained Variation, (b) 1st principal component score, (c) 2nd principal component score, and (d) 3rd principal component score. Figure S4. Average SERS spectra different concentrations of IgG (from 1 to 90 mg/mL). Figure S5. PCA of direct ATP immunoassay: (a) Explained Variation, (b) 1st principal component score, (c) 2nd principal component score, and (d) 3rd principal component score.
