# Supplementary material for: A Direct Immunoassay Based on Surface-Enhanced Spectroscopy Using AuNP/PS-b-P2VP Nanocomposites
Source: Sensors (Basel). 2023 May 16;23(10):4810. doi: 10.3390/s23104810 (PMC10224472; doi:10.3390/s23104810)
Supplement: Supplementary file 1 [file sensors-23-04810-s001.zip › sensors-2358717-supplementary.pdf]

Supporting Information

A Direct Immunoassay Based on Surface-Enhanced Spectroscopy Using AuNP/PS-b-P2VP Nanocomposites

Moyra F. Vieira [1,2], Ana Lívia de Carvalho Bovolato [1], Bruno G. da Fonseca [1], Celly M. S. Izumi [2] and Alexandre G. Brolo [1,*]

[1] Department of Chemistry and Center for Advanced Materials and Related Technologies, University of Victoria, P.O. Box 3065, Victoria, BC V8W 3V6, Canada
[2] Departamento de Química, Instituto de Ciências Exatas, Universidade Federal de Juiz de Fora, Campus Universitário s/n, CEP, Juiz de Fora 36036-900, Brazil
* Correspondence: agbrolo@uvic.ca

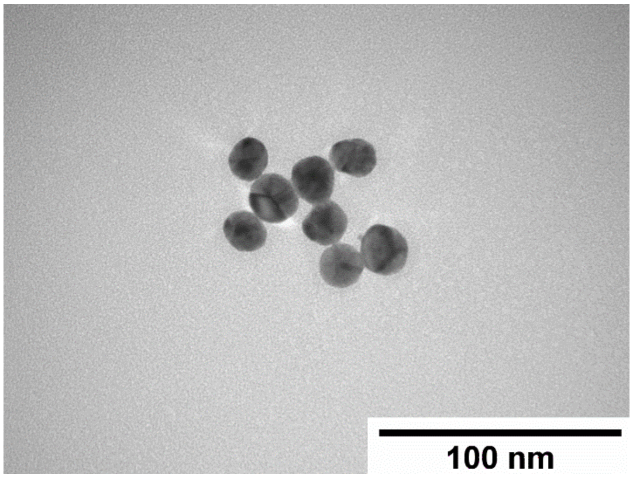

Figure S1. TEM image of the synthesized citrate stabilized gold NPs.

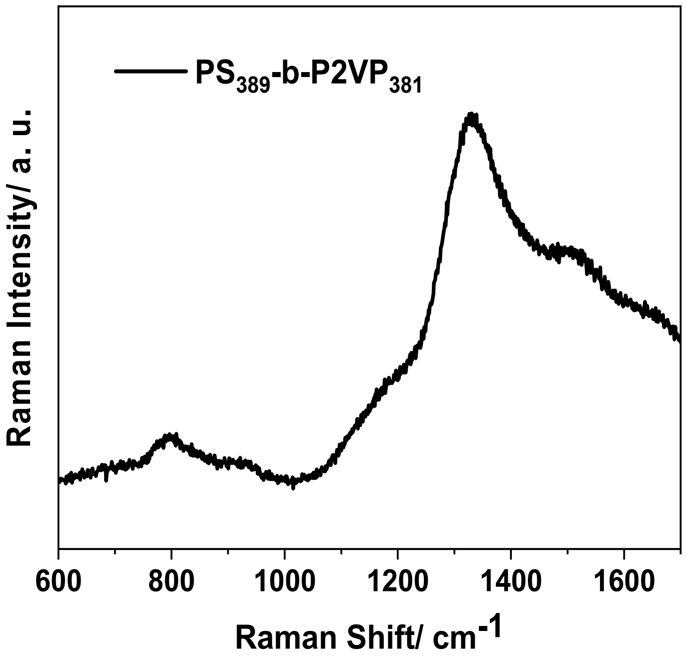

Figure S2. Raman spectra of the PS$_{1250}$-*b*-P2VP$_{1285}$ thin film.

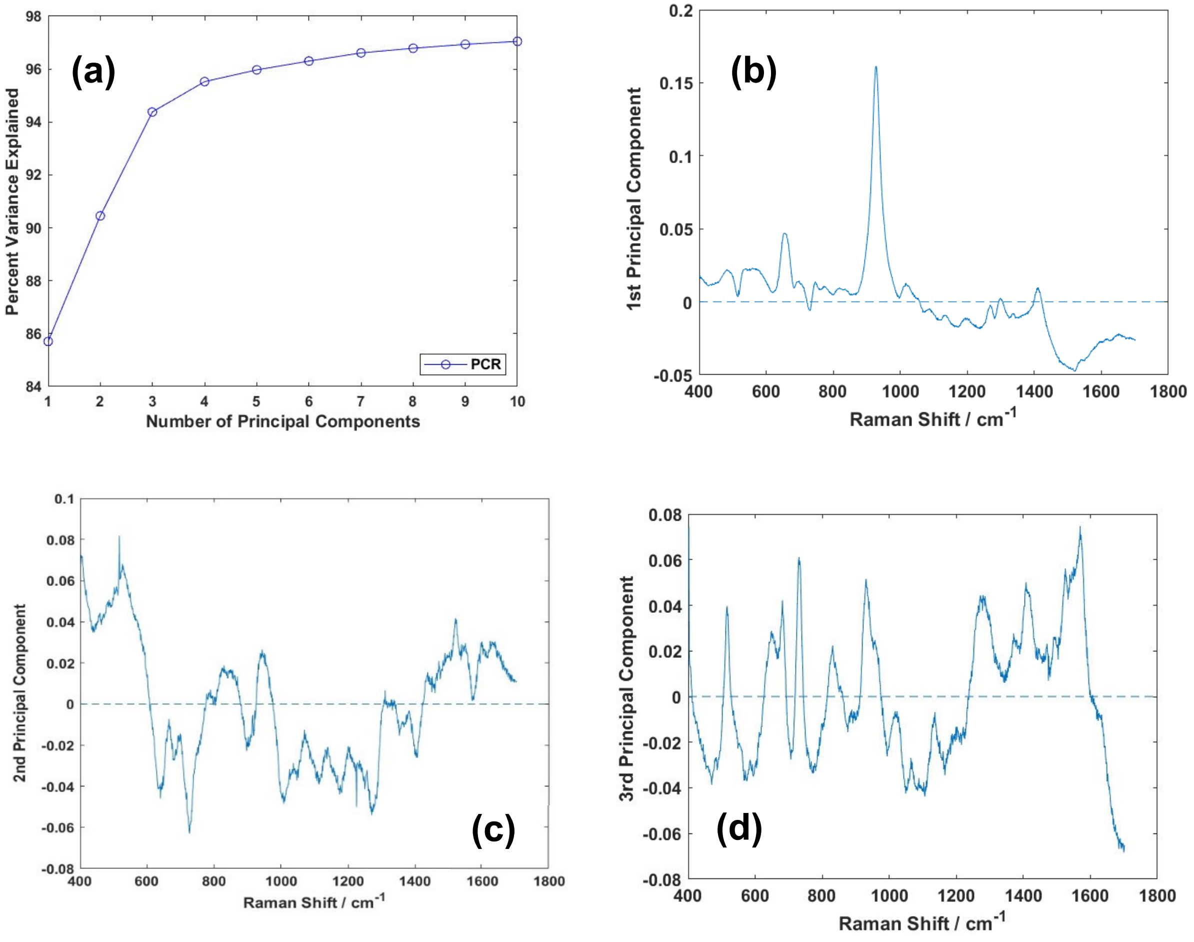

Figure S3. PCA of functionalization steps and IgG detection: (a) Explained Variation, (b) 1st principal component score, (c) 2nd principal component score, (d) 3rd principal component score.

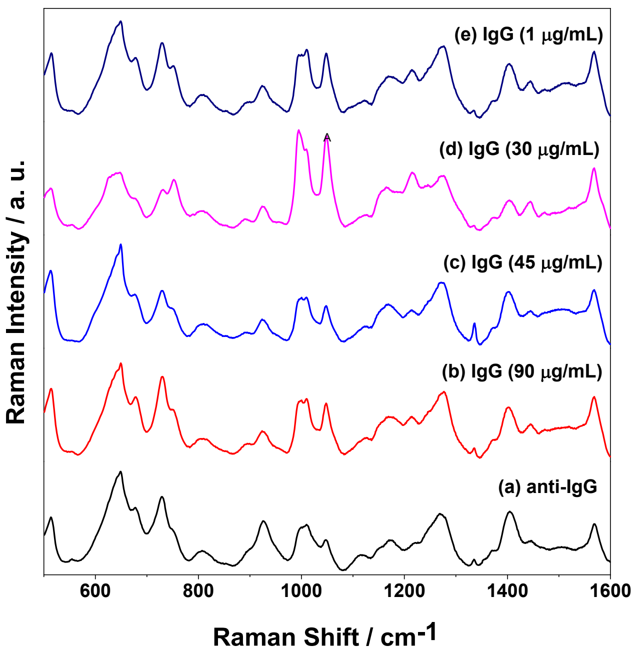

Figure S4. Average SERS spectra different concentrations of IgG (from 1 to 90 μg/mL).

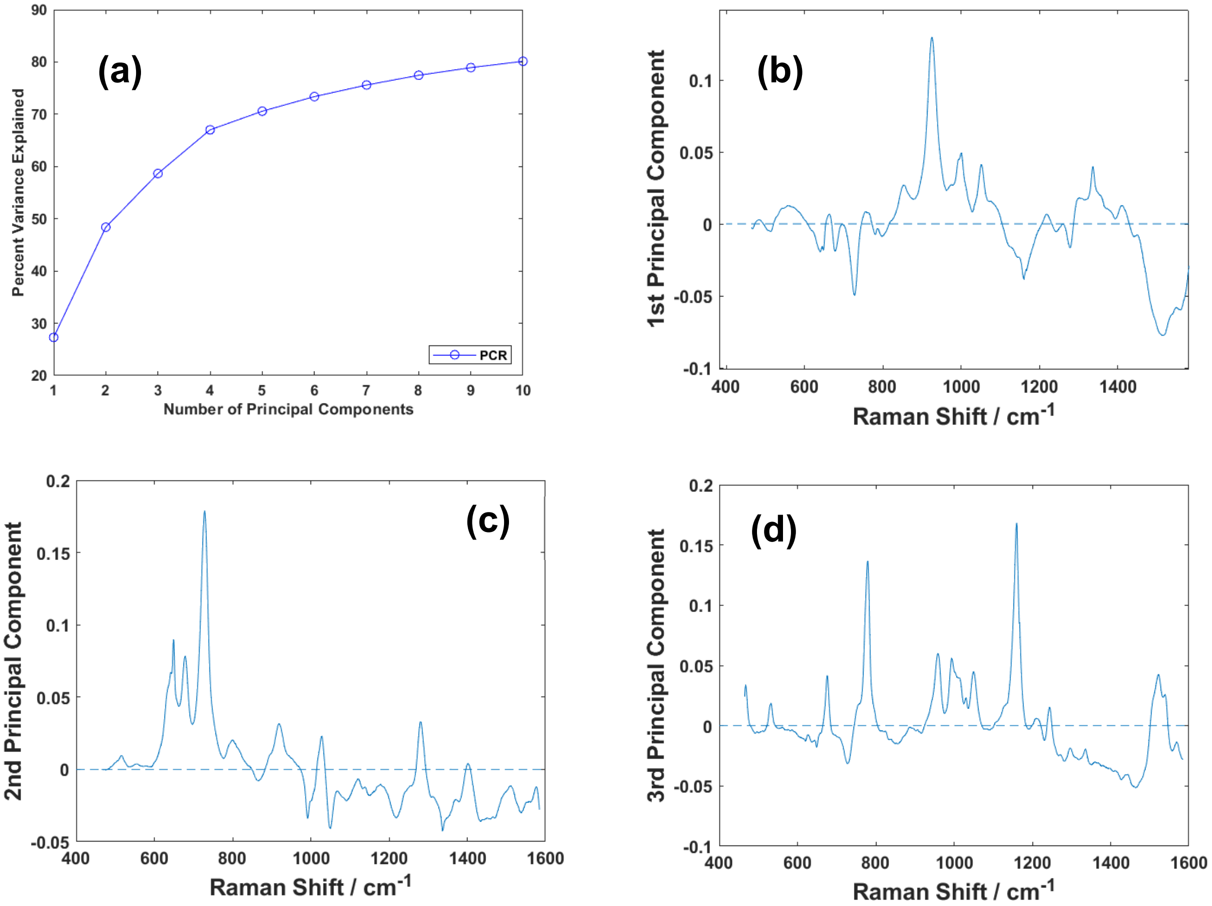

Figure S5. PCA of direct ATP immunoassay: (a) Explained Variation, (b) 1st principal component score, (c) 2nd principal component score, (d) 3rd principal component score.
